# Manipulation of Whitefly Behavior by Plant Viruses

**DOI:** 10.3390/microorganisms10122410

**Published:** 2022-12-06

**Authors:** Kai Zhao, Shu-Sheng Liu, Xiao-Wei Wang, Jin-Guang Yang, Li-Long Pan

**Affiliations:** 1 State Key Laboratory of Rice Biology, Ministry of Agriculture Key Lab of Molecular Biology of Crop Pathogens and Insects, Key Laboratory of Biology of Crop Pathogens and Insects of Zhejiang Province, Institute of Insect Sciences, Zhejiang University, Hangzhou 310058, China; 2 Key Laboratory of Tobacco Pest Monitoring, Controlling & Integrated Management, Tobacco Research Institute of Chinese Academy of Agricultural Sciences, Qingdao 266101, China; 3 The Rural Development Academy, Zhejiang University, Hangzhou 310058, China

**Keywords:** *Bemisia tabaci*, preference, feeding behavior, begomoviruses, criniviruses, vector manipulation

## Abstract

Whiteflies of the *Bemisia tabaci* complex transmit hundreds of plant viruses belonging to the genera *Begomovirus* and *Crinivirus*, among others. Tripartite interactions of whitefly–virus–plant frequently occur during virus infection and transmission. Specifically, virus transmission-related behavior of whitefly, such as preference and feeding, may be altered by viruses and thus exert significant impacts on the outcome of virus spread and epidemics. Here, we provide an overview on the current understanding of the manipulation of whitefly behavior by plant viruses. Plant viruses can significantly modulate whitefly preference and feeding behavior, either directly or in a plant-mediated manner. In general, non-viruliferous whiteflies tend to prefer virus-infected plants, and viruliferous whiteflies are more likely to prefer uninfected plants. In most cases, virus infection of plants and/or whitefly seems to exhibit positive or no effects on whitefly feeding on plants. The significance and evolution of these patterns are then discussed. Finally, we suggest several future directions of research, such as the exploration of temporal dynamics and the dissection of underlying mechanisms of virus-induced changes in whitefly behavior.

## 1. Introduction

Plant viruses cause devastating diseases in a wide range of crops worldwide, resulting in tremendous economic losses annually [1,2]. The majority of plant viruses are transmitted by hemipteran insects such as whiteflies, aphids and planthoppers [3,4], and thus insect vectors play a pivotal role in the transmission and spread of these plant viruses [5,6,7,8]. During virus infection and transmission, active insect vector–virus–plant tripartite interactions frequently occur and serve as important determinants of virus spread and epidemics [9,10,11]. For example, manipulation of the life history of insect vectors by plant viruses is common in nature and bears great significance in determining virus epidemiology [12]. Plant viruses may dramatically modulate the synthesis and signaling of phytohormones, thereby impacting the performance and population dynamics of insect vectors [12,13]. In recent years, studies showed that viruses may significantly modulate the behavior of insect vectors and in turn, affect virus transmission and viral disease incidence (e.g., [14,15,16,17]).

In a productive transmission event of vector-borne plant viruses, insect vectors need to acquire viruses from infected plants through feeding and then disperse to uninfected plants whereon vectors secrete viruses and accomplish virus inoculation [3,18]. During this process, the host plant preference and feeding of insect vectors are behavioral elements of particular relevance to virus transmission. The requirement for insect vectors to feed first on virus-infected plants and then translocate to uninfected plants indicates that the preference of insect vectors is of paramount importance in completing virus transmission. Likewise, feeding is a key behavior of insect vectors in viral spread as both virus acquisition and inoculation entail feeding through stylets. Mathematical modelling and analyses of case studies suggest that any alteration of insect vector preference and feeding behavior may have significant epidemiological consequences [11,19]. Many plant viruses have been shown to manipulate the behavior of insect vectors, thereby significantly impacting viral spread [9,10,12,20]. Such manipulations include both direct effects, wherein the vectors behave differently after virus acquisition, and plant-mediated indirect effects [9,10,11].

The whitefly *Bemisia tabaci* (Gennadius) (Hemiptera: Aleyrodidae), also known as the sweet potato whitefly, has a worldwide distribution and is now known as a complex containing > 44 cryptic species [21,22,23]. These cryptic species are morphologically indistinguishable but genetically divergent and reproductively isolated ([22]; but see [24]). In recent decades, some species of this whitefly complex have emerged as major crop pests in many regions of the world, such as the two invasive whitefly pests, Middle East-Asia Minor 1 ((MEAM1), formerly known as the “B biotype”) and Mediterranean ((MED), formerly known as the “Q biotype”), and some of the African cassava whiteflies such as the Sub-Saharan Africa 1 (SSA1) and the Sub-Saharan Africa 2 (SSA2) [6,7,21,25]. These whitefly pests not only damage crops by direct feeding but also facilitate the prevalence of many economically important crop viral diseases, such as tomato yellow leaf curl, cassava mosaic and cotton leaf curl [8,26]. Whitefly-borne viruses include begomoviruses (*Geminiviridae*), criniviruses (*Closteroviridae*), torradoviruses (*Secoviridae*), ipomoviruses (*Potyviridae*) and carlaviruses (*Betaflexiviridae*). Among these viruses, begomoviruses are transmitted in a circulative manner [27,28]. Criniviruses, ipomoviruses, and torradoviruses are transmitted in a semipersistent manner [27]. Carlaviruses are transmitted in a nonpersistent manner [29].

The increasing economic significance of whitefly and whitefly-borne crop diseases have prompted many studies investigating the direct and indirect effects of plant viruses on whitefly behaviors in recent years. We thus collate available reports and provide an overview on the current understanding of the manipulations of whitefly behavior by plant viruses. We first summarize the plant-mediated and direct manipulations of whitefly preference by plant viruses. We then present how plant viruses directly and indirectly manipulate whitefly feeding. Further, we elaborate on issues of particular relevance to this topic. Finally, we speculate on research directions in the future.

## 2. Manipulation of Whitefly Preference by Plant Viruses

In the life cycle of vector-borne viruses, only when insect vectors land on virus-infected host plants can the viruses be ingested by their vectors. Once insect vectors ingest a certain number of virions, only when they move towards uninfected plants at various distances can plant viruses be transmitted [18]. Hence, temporal and spatial variations in host selection by insect vectors are directly linked to the viral life cycle and determine how extensively they would disseminate.

### 2.1. Single Infections

#### 2.1.1. Plant-Mediated Effects

So far, 36 case studies have been conducted to examine the plant-mediated effects of virus infection on whitefly preference, namely the preference of non-viruliferous whiteflies for virus-infected versus uninfected plants. For viruses of the genus *Begomovirus* (family *Geminiviridae*), 17 case studies have been reported, among which 12 indicated whitefly preference for virus-infected plants, 3 indicated whitefly preference for uninfected plants and 2 indicated no preference (Table 1). For viruses of the genus *Crinivirus* (family *Closteroviridae*), 11 case studies have been reported, among which 6 indicated whitefly preference for virus-infected plants, 3 indicated whitefly preference for uninfected plants and 2 indicated no preference (Table 1). For viruses of the genus *Ipomovirus* (family *Potyviridae*), five case studies have been reported, among which one indicated whitefly preference for virus-infected plants, three indicated whitefly preference for uninfected plants and one indicated no preference (Table 1). For viruses of the genus *Tospovirus* (family *Bunyaviridae*), two reports are available showing whitefly preference for virus-infected plants (Table 1). For the viruses of the genus *Cucumovirus* (family *Bromoviridae*), only one report is available, showing whitefly preference for uninfected plants (Table 1).

Among the begomoviruses described above, TYLCV was the most often examined. Specifically, the majority of studies indicated that non-viruliferous whiteflies preferred TYLCV-infected over uninfected plants [16,30,31,32,33,34,35]. In the case of TYLCV, plant-mediated effects on whitefly preference might be affected by the duration of virus infection. For example, no significant preference of non-viruliferous MEAM1 whiteflies to TYLCV-infected versus uninfected tomato plants was found when plants were at three and 12 weeks post inoculation; however, when plants were at six weeks post inoculation, whiteflies significantly preferred TYLCV-infected plants over uninfected plants [32]. In addition, virus manipulation of vector preference may differ between whitefly species. For instance, while virus-free MED whiteflies preferred TYLCV-infected over uninfected tomato plants, MEAM1 whiteflies preferred uninfected over TYLCV-infected tomato plants [31].

For criniviruses, tomato chlorosis virus (ToCV) is the most often examined. Out of the eight case studies with ToCV, in four studies whiteflies preferred virus-infected over uninfected plants [36,37], in one study whiteflies preferred uninfected over virus-infected plants [38] and in three studies whiteflies exhibited no preference [35,39,40].

For ipomoviruses, the five case studies available were all conducted with squash vein yellowing virus and MEAM1 whitefly, yet the outcome varied with plant species as well as the duration post infection. While MEAM1 whiteflies significantly preferred virus-infected squash plants over uninfected plants, they showed a significant preference for uninfected creeping cucumber plants over plants infected by the virus [41,42]. When the observations were executed with watermelon plants, MEAM1 whiteflies significantly preferred the uninfected plants over infected plants at 9–10 or 10–12 days post viral inoculation, but exhibited no preference when the virus-infected plants were at 5–6 days post inoculation [42,43].

In addition to whitefly-borne plant viruses, other viruses may also impact whitefly preference in a plant-mediated manner. MED whiteflies preferred *Datura stramonium* and pepper plants infected by thrips-transmitted tomato spotted wilt virus (family *Bunyaviridae*, genus *Tospovirus*) over uninfected plants, even though whitefly performance was reduced on virus-infected plants [44]. MEAM1 whiteflies preferred uninfected chili plants over plants infected by aphid-borne cucumber mosaic virus (family *Bromoviridae*, genus *Cucumovirus*) [45].

**Table 1:** Plant-mediated effects of virus infection on whitefly Preference.

Viral Genus	Virus-Isolate	Host Plant	Whitefly	Preference of Virus-Infected versus Uninfected Plants by Non-Viruliferous Whiteflies *	Reference
*Begomovirus*	Tomato yellow leaf curl China virus-Y10	*Nicotiana tabacum* cv. NC89	MEAM1	Virus-infected plants	[46]
	Tomato yellow leaf curl virus-SH2	*Datura stramonium* cv. unknown	MED	Virus-infected plants	[30]
	Tomato yellow leaf curl virus-SH2	*Solanum lycopersicum* cv. Zhongza9	MED	Virus-infected plants	[31]
	Tomato yellow leaf curl virus-unknown	*S. lycopersicum* cv. Florida 47	MEAM1	Virus-infected plants	[32]
	Tomato yellow leaf curl virus-unknown	*S. lycopersicum* cv. Security	MEAM1	Virus-infected plants	[32]
	Tomato severe rugose virus-unknown	*S. lycopersicum* cv. Santa Clara	MEAM1	Virus-infected plants	[39]
	Tomato yellow leaf curl virus-unknown	*S. lycopersicum* cv. Florida 47 R	MEAM1	Virus-infected plants	[34]
	Tomato yellow leaf curl virus-unknown	*S. lycopersicum* cv. Florida 47	MEAM1	Virus-infected plants	[33]
	Tomato yellow leaf curl virus-SH2	*S. lycopersicum* cv. Moneymaker	MED	Virus-infected plants	[16]
	Tomato yellow leaf curl virus-SH2	*N. benthamiana* cv. unknown	MED	Virus-infected plants	[16]
	Chili leaf curl virus-unknown	*Capsicum annum* cv. IIHR 3909	MEAM1	Virus-infected plants	[17]
	Tomato yellow leaf curl virus-Israel	*S. lycopersicum* cv. Moneymaker	MED	Virus-infected plants	[35]
	Tomato yellow leaf curl virus-SH2	*S. lycopersicum* cv. Zhongza9	MEAM1	Uninfected plants	[31]
	Tomato severe rugose virus-unknown	*S. lycopersicum* cv. Santa Clara	MEAM1	Uninfected plants	[38]
	Cucurbit leaf crumplevirus-unknown	*Cucurbita pepo* cv. Goldstar	MEAM1	Uninfected plants	[33]
	Tomato yellow leaf curl virus-unknown	*S. lycopersicum* cv. Florida 47	MEAM1	No preference	[32]
	Tomato yellow leaf curl virus-unknown	*S. lycopersicum* cv. Security	MEAM1	No preference	[32]
*Crinivirus*	Tomato chlorosis virus-unknown	*S. lycopersicum* cv. Zhongza9	MEAM1	Virus-infected plants	[36]
	Tomato chlorosis virus-unknown	*S. lycopersicum* cv. Zhongza9	MED	Virus-infected plants	[36]
	Tomato chlorosis virus-unknown	*S. tuberosum* cv. Agata	MEAM1	Virus-infected plants	[37]
	Tomato chlorosis virus-unknown	*S. tuberosum* cv. Bach-4	MEAM1	Virus-infected plants	[37]
	Cucurbit yellow stunting disorder virus-unknown	*Cucumis melo* cv. Iroquois	MEAM1	Virus-infected plants	[47]
	Cucurbit chlorotic yellows virus-unknown	*C. sativus* cv. Bojie-107	MED	Virus-infected plants	[48]
	Tomato chlorosis virus-unknown	*S. lycopersicum* cv. Santa Clara	MEAM1	Uninfected plants	[38]
	Cucurbit yellow stunting disorder virus-unknown	*C. pepo* cv. Goldstar	MEAM1	Uninfected plants	[33]
	Tomato chlorosis virus-unknown	*S. lycopersicum* cv. Santa Clara	MEAM1	No preference	[39]
	Tomato chlorosis virus-unknown	*S. lycopersicum*cv. Zhongza 9	MED	No preference	[40]
	Tomato chlorosis virus-Pl-1-2	*S. lycopersicum* cv. Moneymaker	MED	No preference	[35]
*Ipomovirus*	Squash vein yellowing virus	*C. pepo* cv. unknown	MEAM1	Virus-infected plants	[42]
	Squash vein yellowing virus	*Melothria pendula* cv. unknown	MEMA1	Uninfected plants	[41]
	Squash vein yellowing virus	*Citrullus lanatus* cv. un Mickylee	MEAM1	Uninfected plants	[42]
	Squash vein yellowing virus	*C. lanatus* cv. Mickylee	MEAM1	Uninfected plants	[43]
	Squash vein yellowing virus	*C. lanatus* cv. Mickylee	MEAM1	No preference	[43]
*Tospovirus*	Tomato spotted wilt virus-unknown	*D. stramonium* cv. unknown	MED	Virus-infected plants	[44]
	Tomato spotted wilt virus-unknown	*C. annuum* cv. Zhongjiao 6	MED	Virus-infected plants	[44]
*Cucumovirus*	Cucumber mosaic virus-unknown	*C. annuum* cv. kulai	MEAM1	Uninfected plants	[45]

* When whitefly preference was tested for different durations, the final choices of whitefly were presented; when multiple experiments were conducted for the same experimental materials, the results from the experiments that resembled natural conditions (i.e., both visual and olfactory stimuli were considered) were presented.

#### 2.1.2. Direct Effects

So far, 27 case studies have been conducted to examine the preference of viruliferous whiteflies to virus-infected versus uninfected plants. For viruses of the genus *Begomovirus*, 19 case studies have been reported, among which 11 studies indicated preference of viruliferous whiteflies for uninfected plants, 2 studies indicated preference for virus-infected plants and 6 studies indicated no preference (Table 2). For viruses of the genus *Crinivirus*, eight case studies have been reported, among which three studies indicated preference of viruliferous whiteflies for uninfected plants, one study indicated preference for virus-infected plants and four studies indicated no preference (Table 2).

Among studies with begomoviruses, all three kinds of results could be found in TYLCV-related studies. For example, while Gautam et al. (2020) showed that TYLCV-infected MEAM1 whiteflies preferred uninfected over TYLCV-infected tomato plants, Fang et al. (2013) reported that both viruliferous MEAM1 and MED whiteflies exhibited no preference for TYLCV-infected or control tomato plants. The genotype of the test plant may significantly impact on the preference of viruliferous whiteflies. While TYLCV-infected MEAM1 whiteflies preferred TYLCV-infected tomato plants of the genotype Florida 47 over uninfected controls, no significant preference was found when tomato plants of the genotype Security were tested [32].

For ToCV-related studies, we found that distinct outcomes could be observed when different whitefly species were used. While ToCV-carrying MEAM1 whiteflies were unable to discriminate ToCV-infected and uninfected tomato plants, viruliferous MED whiteflies exhibited significant preference for uninfected plants over control plants [36].

### 2.2. Mixed Infections

While mixed infection of multiple viruses is common in nature [50], little is known regarding the effects of mixed infections on whitefly preference. So far only three case studies have been reported. Ban et al. (2021) reported that non-viruliferous MEAM1 whiteflies exhibited a significant preference for tobacco plants infected by TYLCV and/or tomato yellow leaf curl China virus over uninfected plants, but no preference was observed between mixed-infected and singly-infected plants [51]. Similarly, Gautam et al. (2020) found that both non-viruliferous and viruliferous whiteflies (infected by cucurbit leaf crumple virus and/or cucurbit yellow stunting disorder virus) preferred uninfected plants over singly- and mixed-infected plants. Further, whiteflies that were infected by TYLCV and/or cucurbit leaf crumple virus, exhibited similar preference for uninfected squash and tomato plants over virus-infected counterparts [33]. These observations suggest that the mixed infection of whitefly and/or plants did not differ from single infection when whitefly preference was concerned.

## 3. Manipulation of Whitefly Feeding Behavior by Plant Viruses

As a group of phytophagous insects, whiteflies rely on feeding to acquire nutrients to support growth and development. Moreover, because whiteflies act as vectors for hundreds of plant viruses, their feeding behavior directly affects viral transmission and by extension, disease epidemiology in crops [18]. Modulation of whitefly feeding behavior is thus of broad ecological significance as it may significantly impact the life history of all three kinds of organisms involved. In theory, plant viruses may manipulate whitefly feeding behavior either via the plant or directly through their action on whitefly physiology. A number of studies have been conducted in this regard, all with single infections.

### 3.1. Plant-Mediated Effects

So far, seven case studies have been conducted to examine the plant-mediated effects of viral infection on whitefly feeding, namely the effects of viral infection of host plants on the feeding of non-viruliferous whiteflies. Because the whitefly is a phloem feeder, to collate results from different studies, here we refer to the total duration of sap ingestion as the index of whitefly feeding behavior. For viruses of the genus *Begomovirus*, three case studies have been reported, and no significant effect was found for all these studies (Table 3). For viruses of the genus *Crinivirus*, two case studies have been conducted, among which, one case indicated promotion of whitefly feeding and one case suggested no effect (Table 3). For viruses of the genus *Ipomovirus*, there were two case studies showing no indirect effects of squash vein yellowing virus infection on whitefly feeding on watermelon plants (Table 3).

### 3.2. Direct Effects and Combined Function of Direct and Plant-Mediated Effects of Virus Infection

In addition to the host plant-mediated indirect effects, acquisition of plant viruses may affect whitefly feeding behavior. Among all the available studies, there are two types of experimental setups. The first is to compare the feeding behavior of viruliferous and non-viruliferous whiteflies on uninfected host plants, and the second is to compare the feeding behavior of non-viruliferous whiteflies on uninfected plants and viruliferous whiteflies on virus-infected host plants. In the latter setup, the effect of viral infection on whitefly feeding behavior is the combined effect of viral infection via both whitefly and host plants. Therefore, here we refer to the former case as the direct effects of viral infection, and the latter as the combined effects of direct and plant-mediated effects of viral infection. As is shown below, the majority of studies on this topic have investigated the direct effects of viral infection on whitefly feeding behavior; only two studies have been conducted on the combined function of direct and plant-mediated effects [15,54].

As for begomoviruses, five cases indicated promotion of whitefly feeding and five cases suggested no effect (Table 4) [52,54,55,56,57,58]. For instance, Wang et al. (2013) reported that viruliferous whiteflies exhibited increased salivary secretion and sustained feeding behaviors on uninfected tomato plants, and Moreno-Delafuente et al. (2013) showed that TYLCV increased the phloem contacts and feeding frequency of whitefly on uninfected eggplants. Liu et al. (2013) showed that TYLCV infection increased the non-phloem feeding parameters of MEAM1 and MED whiteflies, but did not alter the total duration of sap ingestion. Further studies showed that the direct effect of TYLCV on whitefly feeding behavior may vary from positive to no effect depending on the genotype of host plants. Liu et al. (2017) found that TYLCV infection promoted whiteflies feeding on JA-deficient mutant *spr2* and wildtype Castlemart tomato plants, but not on high-JA biosynthesis *35S::prosys* plants.

As for criniviruses, the direct effect of cucurbit chlorotic yellows virus on whitefly probing and feeding behavior is positive in three cases, negative in one case and has no effect in four cases (Table 4) [14,15]. Cucurbit chlorotic yellows virus altered the feeding behavior of whiteflies in a species- and gender-specific manner [14,15]. For example, the infection of cucurbit chlorotic yellows virus impeded the feeding of MEAM1 females, but did not alter that of MEAM1 males as well as MED males and females, on cucumber plants [15].

The direct and combined effects of viral infection on whitefly behavior may be divergent for the same pathosystem. For example, He et al. (2015) revealed that the combined effect of tomato yellow leaf curl China virus was positive for MEAM1 whiteflies, whereas no direct effects of viral infection on whiteflies feeding was found. Similarly, while cucurbit chlorotic yellows virus infection of male MED whiteflies did not significantly alter their feeding, the simultaneous infection of cucumber plants and whiteflies impeded whitefly feeding [15].

## 4. Perspectives on Variability and Consequences of Virus Manipulation of Whitefly Behavior

While so far only a limited number of case studies are available, some patterns seem to emerge. Here we discuss some issues related to the variability and consequences of viral manipulation of whitefly behavior. These issues should be carefully considered in future investigations.

### 4.1. Variability of the Manipulation of Whitefly Behavior by Plant Viruses

For the manipulation of whitefly behavior by plant viruses, differential results can be found when any one partner of the whitefly–virus–plant combination is replaced at the species or even strain/cultivar level. For example, while non-viruliferous MEAM1 whiteflies preferred uninfected over TYLCV-infected tomato plants, MED whiteflies displayed the opposite preference [31]. Viruliferous MEAM1 whiteflies preferred TYLCV-infected over uninfected tomato plants when the cultivar Florida 47 was used, but no preference was found when the cultivar Security was used [32]. This variability suggests that the manipulation of whitefly behavior by plant viruses is a complex but specific process, and each pathosystem requires experimental investigation in its own right. Moreover, this variability highlights that experimental materials must be carefully selected so as to provide reference for dissecting viral epidemics. There are hundreds of species of begomoviruses, and dozens of species of whiteflies [23,59], but in a specific epidemic only one or several whitefly–virus–plant combinations may play a key role in determining the epidemiological outcome. We thus propose that, when exploring the manipulation of whitefly behavior by plant viruses, the first step is to clarify the species of whitefly, virus and plant involved, and to use these materials for experimentation. Only in this way can the studies of tripartite interactions help to understand viral epidemics.

### 4.2. Effects of Whitefly Preference Manipulation on Virus Dissemination

In nature, vector-borne plant viruses that can manipulate vector preference in ways conducive to viral dissemination will exhibit a selective advantage over the others. In theory, for persistently-transmitted, semi-persistently-transmitted and non-persistently-transmitted plant viruses, virus-infected plants should attract non-viruliferous vectors so that viruses can be acquired by insect vector, and repel viruliferous vectors so that they can visit and transmit viruses to uninfected plants [18]. Previous analyses suggest that this pattern of vector preference manipulation by plant viruses is generally the case [9,10,11].

Here, in our analysis of the preference of non-viruliferous and viruliferous whiteflies, although only a limited number of studies are available, a pattern similar to that above seems to emerge. In 12 out of 17 begomovirus-related studies, 6 out of 11 crinivirus-related studies, 1 out of 5 ipomovirus-related studies and 2 out of 2 tospoviruses-related studies, non-viruliferous whitefly preferred virus-infected plants (Table 1). In 11 out of 19 begomovirus-related studies and 3 out of 8 crinivirus-related studies, viruliferous whitefly preferred uninfected plants (Table 2). For these viruses, such a manipulation of whitefly preference may directly promote viral transmission as whiteflies are more likely to acquire and transmit the viruses. It is interesting to note that, while these viruses diverge significantly in genomic and transmission characteristics, they all seem to evolve towards the same direction, further suggesting that these manipulations are adaptive. Detailed dissection of the factors involved such as the identification of viral and plant factors involved may thus unravel the nature of convergent evolution.

### 4.3. Effects of Plant Viruses on Whitefly Feeding

For plant-mediated effects, currently too few case studies are available and thus it seems too early to summarize the patterns. Nevertheless, it seems that begomoviruses (three out of three cases), criniviruses (one out of two cases) and ipomoviruses (two out of two cases) did not significantly alter the total duration of sap ingestion (Table 3). Sap ingestion is presumably the most important step in whitefly feeding, but the other feeding behaviors may also be of significance in modulating whitefly biology. These knowledge gaps should be filled in the future.

It is well-established that plant viruses may significantly alter the performance of insect vectors such as whiteflies in a plant-mediated manner [9,10,11,60]. The modulation of vector performance is attributed, in most cases, to changes in the synthesis and/or signaling of phytohormones in plants [13,61]. Feeding is directly associated with insect performance; thus, we urge more investigations to examine whether virus-induced changes in the feeding of insect vectors play a role in modulating their performance. Such explorations may unravel the underpinnings of changes in insect vector performance.

For direct effects, it seems that both begomoviruses (5 out of 10 cases) and criniviruses (3 out of 8 cases) tend to promote the feeding of whitefly on uninfected plants (Table 4). For begomoviruses such as TYLCV, previous studies suggest they may be reminiscent of insect pathogens as association with begomoviruses often resulted in reduced whitefly longevity and fertility [62]. This claim is further supported by reports showing that TYLCV may replicate within whitefly [63,64]. The contradiction between whitefly feeding and performance suggests that factors other than feeding manipulation may determine the deleterious direct effects of viruses on whitefly performance.

### 4.4. Evolution of the Manipulation of Whitefly Preference by Plant Viruses

Overview of the manipulation of whitefly preference by plant viruses indicated that non-viruliferous whiteflies are more likely to prefer virus-infected plants, and viruliferous whiteflies are more likely to prefer uninfected plants. In the field, such a manipulation may benefit viral spread substantially as it promoted viral acquisition by non-viruliferous whiteflies and viral inoculation on uninfected plants by viruliferous whiteflies. On this basis, we propose that the manipulation of whitefly preference by plant viruses is driven by and adaptive for viruses. Under natural conditions, plant viruses exist as an ensemble of genetically non-identical but closely related viral individuals due to the error-prone viral replication processes [65]. The large population of viral entities enables rapid adaptive viral evolution as the gain-of-function mutations maybe already present for many kinds of selection pressures. When whitefly vectors are concerned, viruses that can efficiently modulate whitefly preference to promote their own spread may obtain significant advantages in competition and thus outcompete the other viral entities. In the long term, repeated action of selection pressure associated with whitefly transmission may then shape the current genetic landscape of whitefly-borne plant viruses.

## 5. Future Prospective

As described above, a number of interesting investigations have been conducted on the manipulation of whitefly behavior by plant viruses. However, the gaps in our knowledge are still huge, especially in the details and underlying mechanisms. Hence, more studies are needed in the future to uncover more details and clarify more general mechanisms. The issues that are of particular relevance to this review and future endeavors are discussed below.

### 5.1. Temporal Dynamics of the Manipulation of Whitefly Behavior by Plant Viruses

Behavior modulation of insect vectors by plant viruses is a continuous and dynamic process, wherein changes may occur with the progression of time. Thus, behavior modulation of insect vectors needs to be analyzed from the perspective of temporal dynamics. Firstly, it takes time for whiteflies to perceive and then respond to clues from plants. For example, MEAM1 whiteflies exhibited no preference for squash vein yellowing virus-infected versus uninfected creeping cucumber plants within the first 5 h after release, but they significantly preferred uninfected over virus-infected plants at 24 h post release [41]. In addition to the experimental duration, the progression of viral infection in host plants may also impact on the modulation of insect behavior by plant viruses. For instance, viruliferous whiteflies exhibited no preference for cotton leaf curl virus-infected versus uninfected cotton plants when the plants were infected by the virus for less than 5 days; however, when the plants were infected by the virus for 20 or 35 days, whiteflies showed significant preference for the uninfected over virus-infected plants [49].

During the progression of viral infection in insect vectors or host plants, active interactions of virus–insect and virus–plant may occur and lead to significant changes in insect and plant physiology, respectively. Under natural conditions, this situation may occur as well and thus have important ecological implications. Therefore, understanding these temporal dynamics is vital to fully dissect virus epidemics.

### 5.2. The Mechanisms Underlying the Modulation of Whitefly Behavior by Plant Virus

For plant-mediated effects on whitefly preference, reports show that visual cues such as color and olfactory cues such as plant volatiles, may play a major role in mediating the preference of whitefly for virus-infected or uninfected plants [34,39,44,48,66]. For example, infection of tomato plants by tomato severe rugose virus and ToCV-induced leaf chlorosis and yellowing, which significantly attracted whiteflies, led to increased whitefly preference for virus-infected over uninfected plants [39]. In the study of tomato yellow leaf curl China virus, the viral infection inhibited the biosynthesis of whitefly-repelling volatile terpenes by disrupting the jasmonate signaling pathway, thereby making virus-infected plants more attractive to whiteflies than uninfected controls [66,67,68].

For the mechanisms underlying the direct effects of viral infection on whitefly preference, so far only one detailed case study has been reported. It was found that while non-viruliferous MED whiteflies significantly preferred TYLCV-infected plants over uninfected plants, TYLCV-carrying whiteflies exhibited an equal preference for TYLCV-infected and uninfected plants [31]. Mechanistic exploration revealed that when whiteflies acquired TYLCV, the virus caused caspase-dependent apoptotic neurodegeneration with severe vacuolar neuropathological lesions in the brain of whitefly by inducing a putative inflammatory signaling cascade of innate immunity; the sensory defects induced by neurodegeneration abolished whitefly preference for TYLCV-infected plants [16].

For the modulation of whitefly feeding behavior by plant viruses, so far no studies have been conducted to directly examine the underlying mechanisms. This may be due in part to the tiny size of whitefly, which becomes problematic when performing genetic manipulation and then monitoring whitefly feeding. For plant-mediated effects, one of the possible mechanisms might be that viral infection alters the palatability of host plants by modulating the contents of nutrients and defensive chemicals, as seen in some studies [69]. For the effects of viral infection on whitefly feeding, more in-depth studies are needed to unravel the molecular underpinnings.

While valuable progress has been made in the research into the modulation of whitefly preference and feeding by plant viruses, there are still many knowledge gaps that need to be addressed. The first is that so far mechanistic exploration has been conducted in too few pathosystems, making it impossible to draw any universal conclusion. This issue will be better resolved in the future when more in-depth studies using new whitefly-virus–plant combinations are available. The second is that major factors associated with whitefly, virus and plant in the modulation of whitefly behavior remain mostly unexplored. To address this issue, more molecular and genetic tools should be incorporated into experimental studies, as summarized in Ray and Casteel (2022). The last is the role of abiotic factors such as light and temperature, which may significantly modulate plant and insect physiology, and thus potentially the whitefly–virus–plant tripartite interactions. An excellent example is provided by Zhao et al. (2021), wherein the authors explored the impact of red-light on plant-mediated, virus-induced whitefly preference and the molecular mechanisms involved [70]. Similar studies should be conducted and will provide valuable insights into viral epidemics.

## Figures and Tables

**Table 2:** Direct effects of virus infection on whitefly Preference.

Viral Genus	Virus-Isolate	Host Plant	Whitefly	Preference of Virus-Infected versus Uninfected Plants by Viruliferous Whiteflies *	Reference
*Begomovirus*	Tomato yellow leaf curl virus-unknown	*S. lycopersicum* cv. Florida 47	MEAM1	Virus-infected plants	[32]
	Tomato yellow leaf curl virus-Israel	*S. lycopersicum* cv. Moneymaker	MED	Virus-infected plants	[35]
	Cotton leaf curl virus-unknown	*Gossypium hirsutum* cv. F846	Unknown	Uninfected plants	[49]
	Cotton leaf curl virus-unknown	*G. hirsutum* cv. F846	Unknown	Uninfected plants	[49]
	Tomato yellow leaf curl virus-unknown	*S. lycopersicum* cv. Florida 47	MEAM1	Uninfected plants	[32]
	Tomato yellow leaf curl virus-unknown	*S. lycopersicum* cv. Florida 47	MEAM1	Uninfected plants	[32]
	Tomato yellow leaf curl virus-unknown	*S. lycopersicum* cv. Security	MEAM1	Uninfected plants	[32]
	Tomato yellow leaf curl virus-unknown	*S. lycopersicum* cv. Security	MEAM1	Uninfected plants	[32]
	Tomato severe rugose virus-unknown	*S. lycopersicum* cv. Santa Clara	MEAM1	Uninfected plants	[39]
	Tomato severe rugose virus-unknown	*S. lycopersicum* cv. Santa Clara	MEAM1	Uninfected plants	[38]
	Cucurbit leaf crumple virus-unknown	*C. pepo* cv. Goldstar	MEAM1	Uninfected plants	[33]
	Tomato yellow leaf curl virus-unknown	*S. lycopersicum* cv. Florida 47	MEAM1	Uninfected plants	[33]
	Chili leaf curl virus-unknown	*C. annum* cv. IIHR 3909	MEAM1	Uninfected plants	[17]
	Cotton leaf curl virus-unknown	*G. hirsutum* cv. F846	Unknown	No preference	[49]
	Tomato yellow leaf curl virus-SH2	*S. lycopersicum* cv. Zhongza 9	MEAM1	No preference	[31]
	Tomato yellow leaf curl virus-SH2	*S. lycopersicum* cv. Zhongza 9	MED	No preference	[31]
	Tomato yellow leaf curl virus-unknown	*S. lycopersicum* cv. Security	MEAM1	No preference	[32]
	Tomato yellow leaf curl virus-SH2	*S. lycopersicum* cv. Moneymaker	MED	No preference	[16]
	Tomato yellow leaf curl virus-SH2	*N. benthamiana* cv. unknown	MED	No preference	[16]
*Crinivirus*	Cucurbit chlorotic yellows virus-unknown	*C. sativus* cv. Bojie-107	MED	Virus-infected plants	[48]
	Tomato chlorosis virus-unknown	*S. lycopersicum* cv. Zhongza 9	MED	Uninfected plants	[36]
	Tomato chlorosis virus-unknown	*S. lycopersicum*cv. Zhongza 9	MED	Uninfected plants	[40]
	Cucurbit yellow stunting disorder virus-unknown	*C. pepo* cv. Goldstar	MEAM1	Uninfected plants	[33]
	Tomato chlorosis virus-unknown	*S. lycopersicum* cv. Santa Clara	MEAM1	No preference	[39]
	Tomato chlorosis virus-unknown	*S. lycopersicum* cv. Santa Clara	MEAM1	No preference	[38]
	Tomato chlorosis virus-unknown	*S. lycopersicum* cv. Zhongza 9	MEAM1	No preference	[36]
	Tomato chlorosis virus-Pl-1-2	*S. lycopersicum* cv. Moneymaker	MED	No preference	[35]

* When whitefly preference was tested for different durations, the final choices of whitefly were presented; when multiple experiments were conducted for the same experimental materials, the results from the experiments that resembled natural conditions (i.e., both visual and olfactory stimuli were considered) were presented.

**Table 3:** Plant-mediated effects of virus infection on whitefly feeding behavior.

Viral Genus	Virus-Isolate	Host Plant	Whitefly	Plant-Mediated Effects of Virus on Duration of Sap Ingestion by Whiteflies *	Reference
*Begomovirus*	Tomato yellow leaf curl virus-SH2	*S. lycopersicum* cv. Zhongza9	MED	No effect	[52]
	Tomato yellow leaf curl virus-SH2	*S. lycopersicum* cv. Zhongza9	MEAM1	No effect	[52]
	Tomato severe rugose virus-unknown	*S. lycopersicum* cv. Santa Clara	MEAM1	No effect	[53]
*Crinivirus*	Cucurbit yellow stunting disorder virus-unknown	*C. melo* cv. Iroquois	MEAM1	Positive	[47]
	Tomato chlorosis virus-unknown	*S. lycopersicum* cv. Santa Clara	MEAM1	No effect	[53]
*Ipomovirus*	Squash vein yellowing virus-unknown	*C. lanatus* cv. Mickylee	MEAM1	No effect	[43]
	Squash vein yellowing virus-unknown	*C. lanatus* cv. Mickylee	MEAM1	No effect	[43]

* As whiteflies feed on plant phloem sap, total duration of sap ingestion was used as the index of whitefly feeding behavior.

**Table 4:** Direct effects of virus infection on whitefly feeding behavior.

Viral Genus	Virus-Isolate	Host Plant	Whitefly	Direct Effects of Virus on Duration of Sap Ingestion by Whiteflies *	Reference
*Begomovirus*	Tomato yellow leaf curl virus-unknown	*S. lycopersicum* cv. Xianke 6	MED	Positive	[57]
	Tomato yellow leaf curl virus-Israel	*S. melongena* cv. Black Beauty	MED	Positive	[56]
	Tomato yellow leaf curl virus-unknown	*S. lycopersicum* cv. unknown	MED	Positive	[58]
	Tomato yellow leaf curl virus-SH2	*S. lycopersicum* cv. Castlemart wildtype	MEAM1	Positive	[55]
	Tomato yellow leaf curl virus-SH2	*S. lycopersicum* cv. Castlemart *spr2* mutant	MEAM1	Positive	[55]
	Tomato yellow leaf curl virus-SH2	*S. lycopersicum* cv. Zhongza 9	MED	No effect	[52]
	Tomato yellow leaf curl virus-SH2	*S. lycopersicum* cv. Zhongza 9	MEAM1	No effect	[52]
	Tomato yellow leaf curl China virus-Y10	*G. hirsutum* cv. Zhe-Mian 1793	MEAM1	No effect	[54]
	Tomato yellow leaf curl China virus-Y10	*N. tabacum* cv. NC89	MEAM1	No effect	[54]
	Tomato yellow leaf curl virus-SH2	*S. lycopersicum* cv. Castlemart 35S::*prosys*	MEAM1	No effect	[55]
*Crinivirus*	Cucurbit chlorotic yellows virus-unknown	*C. sativus* cv. Lvjian-1	MEAM1	Positive	[14]
	Cucurbit chlorotic yellows virus- unknown	*C. sativus* cv. Lvjian-1	MED	Positive	[14]
	Cucurbit chlorotic yellows virus- unknown	*C. sativus* cv. Lvjian-1	MED	Positive	[14]
	Cucurbit chlorotic yellows virus- unknown	*C. sativus* cv. Bojie-107	MEAM1	Negative	[15]
	Cucurbit chlorotic yellows virus-unknown	*C. sativus* cv. Lvjian-1	MEAM1	No effect	[14]
	Cucurbit chlorotic yellows virus- unknown	*C. sativus* cv. Bojie-107	MEAM1	No effect	[15]
	Cucurbit chlorotic yellows virus- unknown	*C. sativus* cv. Bojie-107	MED	No effect	[15]
	Cucurbit chlorotic yellows virus- unknown	*C. sativus* cv. Bojie-107	MED	No effect	[15]

* As whiteflies feed on plant phloem sap, total duration of sap ingestion was used as the index of whitefly feeding behavior.

## Data Availability

Not applicable.
